# Comparative evaluation of glomerular morphometric techniques reveals differential technical artifacts between focal segmental glomerulosclerosis and normal glomeruli

**DOI:** 10.14814/phy2.15688

**Published:** 2023-07-09

**Authors:** Anand C. Reghuvaran, Qisheng Lin, John M. Basgen, Khadija Banu, Hongmei Shi, Anushree Vashist, John Pell, Sudhir Perinchery, John C. He, Dennis Moledina, F. Perry Wilson, Madhav C. Menon

**Affiliations:** ^1^ Division of Nephrology, Department of Medicine Yale University School of Medicine New Haven Connecticut USA; ^2^ Department of Nephrology, Renji Hospital, School of Medicine Shanghai Jiao Tong University Shanghai People's Republic of China; ^3^ Morphometry and Stereology Laboratory Charles R. Drew University of Medicine and Science Los Angeles California USA; ^4^ Department of Pathology Yale University School of Medicine New Haven Connecticut USA; ^5^ Division of Nephrology, Department of Medicine Icahn School of Medicine at Mount Sinai New York New York USA; ^6^ Clinical Translational Research Accelerator, Department of Medicine Yale University School of Medicine New Haven Connecticut USA

**Keywords:** 3‐Profile method, Cavalieri method, glomerular morphometry, Weibel–Gomez method

## Abstract

Morphometric estimates of mean or individual glomerular volume (MGV, IGV) have biological implications, over and above qualitative histologic data. However, morphometry is time‐consuming and requires expertise limiting its utility in clinical cases. We evaluated MGV and IGV using plastic‐ and paraffin‐embedded tissue from 10 control and 10 focal segmental glomerulosclerosis (FSGS) mice (aging and 5/6th nephrectomy models) using the gold standard Cavalieri (Cav) method versus the 2‐profile and Weibel–Gomez (WG) methods and a novel 3‐profile method. We compared accuracy, bias and precision, and quantified results obtained when sampling differing numbers of glomeruli. In both FSGS and controls, we identified an acceptable precision for MGV of 10‐glomerular sampling versus 20‐glomerular sampling using the Cav method, while 5‐glomerular sampling was less precise. In plastic tissue, 2‐ or 3‐profile MGVs showed greater concordance with MGV when using Cav, versus MGV with WG. IGV comparisons using the same glomeruli reported a consistent underestimation bias with both 2‐ or 3‐profile methods versus the Cav method. FSGS glomeruli showed wider variations in bias estimation than controls. Our 3‐profile method offered incremental benefit to the 2‐profile method in both IGV and MGV estimation (improved correlation coefficient, Lin's concordance and reduced bias). In our control animals, we quantified a shrinkage artifact of 52% from tissue processed for paraffin‐embedded versus plastic‐embedded tissue. FSGS glomeruli showed overall reduced shrinkage albeit with variable artifact signifying periglomerular/glomerular fibrosis. A novel 3‐profile method offers slightly improved concordance with reduced bias versus 2‐profile. Our findings have implications for future studies using glomerular morphometry.

## Graphical abstract

Detailed glomerular morphometry performed on kidney tissue offers advantages over routine histopathology of glomerular diseases. Here we evaluate routinely used morphometric techniques, as well as number of glomeruli sampled, using control and focal segmental glomerulosclerosis kidneys. We show that adding a third section profile to the reported 2‐profile technique marginally improves accuracy and reduces bias of glomerular volume (GV) data with a proportionate increase in operator effort. We also clearly demonstrate that paraffin embedding has differential shrinkage effects on fibrotic tissue, a source of bias that needs to be considered when using GV for diagnoses and prognostication.

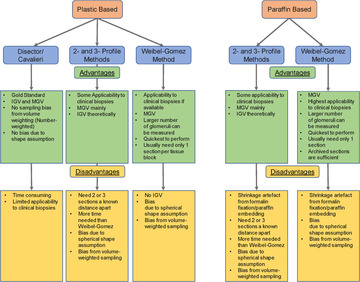

## 1 INTRODUCTION

Glomerular structural evaluation using quantitative analysis has often revealed additional prognostic benefit over qualitative histological analysis in human diseases (Fufaa et al., 2016; Haruhara et al., 2021; Lemley et al., 2016; Merzkani et al., 2021; Nishizono et al., 2017; Puelles et al., 2019; Romoli et al., 2018; Velez et al., 2017). For instance, we and others reported focal segmental glomerulosclerosis (FSGS) was associated with large glomerular volumes (GVs) and podocyte hypertrophy, and large GV predicted outcomes in human nephrotic syndromes (Banu et al., 2021; Lemley et al., 2016). Pathophysiologically, GV may reflect antecedent nephron injury and loss, with the consequent need for remaining glomeruli to hypertrophy to increase the effective filtration surface area (Ellis et al., 1986; Hirose et al., 1980). Excessive or disproportionate glomerular hypertrophy with respect to podocyte hypertrophy promoted podocyte loss, glomerulosclerosis and progressive chronic kidney disease (Nishizono et al., 2017; Puelles et al., 2019). Thus, GV estimation has diagnostic and prognostic implications in human disease and in human and animal research (Naik et al., 2021; Walker et al., 2012).

When planning a quantitative study to estimate GV, several factors need to be considered such as prospective measuring methods, sampling protocols, embedding medium, magnification of images and the time anticipated to perform the study, all of which may influence study results. The accuracy and precision of the different measuring methods will also affect the optimal number of glomeruli to be measured and ought to be considered.

The gold standard technique for GV estimation is the Cavalieri (Cav) method. The method uses the disector principle to select glomeruli for measurement and is independent of size and shape (Gundersen & Jensen, 1987; Najafian et al., 2002; Sterio, 1984). Individual GV (IGV‐Cav) are measured providing a distribution of the IGVs and can be used to calculate the mean GV (MGV‐Cav). The method requires multiple sections (~6), a known distance apart, throughout the individual glomeruli, making it a time‐intensive technique. Only complete glomeruli can be used for measurement. The 2‐profile (2P) method (Najafian et al., 2002) and the 3‐profile (3P) method introduced here use two or three sections through individual glomeruli and assume glomeruli are spheres which will add a bias to the estimate of GV. The Weibel–Gomez (WG) method uses a single section through individual glomeruli (Weibel, 1979) and is only used to measure the mean glomerular volume (MGV‐WG). The 2P, 3P, and WG methods typically do not use disector sampling but use single section sampling that will decrease the time needed to cut sections and select glomeruli for measurement but may add a sampling bias to the GV estimates.

A typical paraffin‐embedded renal biopsy archived for retrospective studies includes limited numbers of glomeruli. In a recent study (Zhang et al., 2021) we reported that a subset of paraffin‐embedded biopsy tissue from the NEPTUNE consortium (Sampson et al., 2016) had a mean ± SD of 14 ± 10 (median 12) glomerular profiles. Prior data suggest that using MGV‐Cav from >10 glomeruli was not associated with significant improvements in the coefficient of variation (CV) while calculating MGV. Whether these sample sizes are also applicable to paraffin‐based methods is not clear from current literature. Similarly, whether less time‐intensive morphometric techniques performed in plastic‐embedded tissue offer comparable precision to the GV‐Cav is also unknown. A quantification of whether and how structural glomerular pathology (e.g., FSGS) influences MGV measurements in paraffin‐ or plastic‐embedded tissue has also not been previously performed.

Based on the significance of GV measurements to glomerular disease, we undertook a detailed evaluation of techniques measuring MGV and IGV in mice. To do this, we used two murine models of FSGS as well as controls to compare three previously published methods for measuring MGV the Cav, 2P and WG methods. In addition, we introduced a novel 3P method where we tested the addition of a third glomerular profile to the 2P method and evaluated benefit in IGV and MGV measurements when compared with the Cav method. Using plastic‐embedded tissue, we also studied the number of glomeruli vis‐à‐vis precision in reflecting MGV by applying a modeling strategy that assumes random sampling of glomeruli to the gold standard GV‐Cav estimate. We documented shrinkage artifact (Dorph‐Petersen et al., 2001) introduced by formalin fixation and paraffin embedding that is present regardless of the individual techniques tested. Surprisingly, the presence of FSGS reduced this shrinkage artifact albeit non‐uniformly, possibly reflecting the variable presence of glomerular fibrosis in FSGS kidneys. The data here are informative for future experimental morphometric studies.

## 2 MATERIALS AND METHODS

### 2.1 Factors affecting glomerular volume measurements

To determine the most efficient method to measure individual and mean GV several factors were evaluated: (1) embedding medium: plastic versus paraffin; (2) measuring method: Cav, 2‐profile, 3‐profile, and WG; (3) magnification: 100× lens versus 40× lens; (4) normal glomeruli versus FSGS; (5) number of glomeruli.

### 2.2 Animals

We restricted our analyses to BALBc/J mice to avoid any strain‐related variation in comparisons and used young male adult BALBc/J as controls (14–20 weeks of age) for evaluating baseline GV (*n* = 10). To evaluate morphometry in kidneys with significant glomerular injury, we used male BALBc/J mice from two FSGS models (*n* = 10) as reported (Banu et al., 2021; Wei et al., 2018). Model 1 was a 1/6‐remnant kidney model of FSGS (Banu et al., 2021; Tan et al., 2019). Briefly, at 8–10 weeks of age, two‐thirds of the left kidney was removed, and 1 week later the right kidney was removed. Six weeks after right nephrectomy, (i.e., age 15–17 weeks) the mice were anesthetized with ketamine (#40027676, Zoetis Inc.)/xylazine (#NDC59399‐110‐20, Akorn Inc.) mixture (intraperitoneally) and perfused with PBS via cardiac perfusion (25G butterfly canula, BD Biosciences) and the remnant kidney processed for morphometric analysis. A nick was made in the inferior vena cava below the renal veins using a 22G needle before initiation of perfusion, and perfusion was continued till kidney showed pearly white coloration, and colorless perfusate drained from the nick. The perfusion rate was set constant for all animals, at 8.3 mL/min using a Cytiva P1 peristaltic pump. This ensured that the flow pressure was equal across all samples and would not have altered the glomerular size significantly. Model 2 was an aging model of FSGS we reported in mice with inducible Shroom3 knockdown (Banu et al., 2021; Wei et al., 2018). At 12 months of age, mice were fed doxycycline for 8 weeks to induce Shroom3 knockdown, podocyte effacement, followed by podocyte loss, proteinuria, azotemia, and FSGS lesions. FSGS mice were perfused with PBS via cardiac perfusion and the left kidney processed for morphometric analysis. The FSGS lesions in these mice models were reported before and confirmed and characterized by a renal pathologist. Male BALBc/J mice aged 14–20 weeks were sacrificed and used as controls of normal glomerular structure (*n* = 10). The cannulation, perfusion, and sample procedures were uniform across FSGS and control animals.

### 2.3 Embedding protocols

#### 2.3.1 Plastic embedment

Kidney cortical specimens were cut into 1‐mm cubes, fixed in 1% glutaraldehyde, post‐fixed in 1% osmium tetroxide, dehydrated through an ethanol series, and embedded in Poly/Bed 812 (Polysciences Inc.). Sections were cut using a Reichert‐Jung Ultracut E ultramicrotome (Leica Biosystems) set to cut 1‐μm thick sections. Section thickness was validated by embedding a section in Poly/Bed 812, cutting an ultrathin (silver‐gold) section perpendicular to the original section plane, imaging the edge of the original section using an electron microscope, and measuring the thickness of the section. For each animal, a plastic block was arbitrarily selected, trimmed, and the first technically good section was saved to a slide, stained with 1% toluidine blue, coverglassed, and labeled Section 0. With the ultramicrotome continuously cutting, every 10th section was saved to a slide and numbered sequentially 10, 20, 30 until the 200th section was saved (21st slide). Slides were then stained and coverglassed.

#### 2.3.2 Paraffin embedment

Kidneys were bisected, submerged in 4% formaldehyde, dehydrated through an ethanol series, and embedded in paraffin. Sections were cut using a standard microtome set to cut 5‐μm thick sections. A series of five adjacent sections were saved to slides sequentially numbered 1, 2, 3, 4, and 5, stained with PAS, and coverglassed.

### 2.4 Sampling and imaging protocols

Glomeruli are three‐dimensional particles. When sections are cut and imaged, three‐dimensional glomeruli are not present in the sections, two‐dimensional profiles (samples) from the glomeruli are present and available to be imaged and measured. This is an important distinction when performing quantitative studies.

A fundamental step when measuring glomeruli is to select the glomeruli to be measured. A frequently used sampling technique is to arbitrarily select a single section and measure the glomeruli in that section. This single‐section sampling technique also known as volume‐weighted sampling is biased because large glomeruli have a greater probability of intersecting the section than small glomeruli (Gundersen, 1986). To overcome this sampling bias, Sterio introduced the disector sampling tool in 1984 (Sterio, 1984). A disector is a pair of sections a known distance apart. This distance between the sections cannot be greater than the smallest possible glomerular diameter in the kidney. Only glomeruli intersected by the second section of the pair but not the first section are selected for measurement (Figure S1). The disector sampling tool is an integral part of the Cav method. Normally the single‐section sampling technique is used to select glomeruli for the 2P, 3P, and WG methods and was used to select glomeruli for the MGV comparisons. For this comparison study in order to make IGV comparisons, the exact same glomeruli were selected with the disector for all four methods.

A microscope (Carl Zeiss Microscopy) fitted with a digital camera (Jenoptix AG) connected to an iMAC computer with 27″ monitor (Apple Inc.) was used for imaging. Photoshop software (Adobe Inc.) was used to observe the images and montage the images into a single image for each section.

#### 2.4.1 Plastic sections

The 10× microscope lens was utilized to image each of the 21 saved sections from a kidney. Sections 0 and 10 were observed simultaneously as a disector pair. Newly appearing glomeruli in Section 10 but not present in Section 0 were noted and their profiles numbered sequentially (1, 2, 3, … *x*) (Figure S1). Next, the 10× images from Sections 10 and 20 were observed simultaneously. Profiles from glomeruli numbered in Section 10 and present in Section 20 were numbered with their same numbers. Newly appearing glomeruli in Section 20 were then noted and their profiles numbered (*x* + 1, *x* + 2, etc.). This was repeated until a total of 20 glomeruli were identified and their profiles appropriately numbered in each of the sections they appeared in. Finally, using the labeled 10× images to identify the numbered glomeruli, the profiles from each glomerulus from each section were imaged with the 100× lens. These stacks of images were used to measure individual GV with the Cav method (Figure S2). For the 2P method, the stack of images obtained for the Cav method was available. A random number generator was used to determine the first image from the stack to be used. Then the next image in the stack was necessarily used (Figure S2). This was repeated for all 20 glomeruli. For the 3P method the next adjacent image in the stack was necessarily used (Figure S2). For the WG method a random number generator was used to determine which single image from the stack was used from each glomerulus (Figure S2).

#### 2.4.2 Paraffin sections

The Cav method was not tested in paraffin sections. The single‐section sectioning protocol was used to select glomeruli and the same glomeruli were not necessarily measured using the three methods. The 10× lens was used to image several adjacent fields of the cortical region of Section 1. The images were montaged together using Photoshop. The same region of the kidney was imaged, and the images montaged for Sections 3 and 5. For the 2P method the images from Sections 1 and 3 were observed simultaneously using Photoshop. Glomeruli present in both sections were noted and their profiles sequentially numbered from 1 to 20. Using the 100× lens and the labeled images, the profiles from the 20 glomeruli on both sections were imaged. For the 3P method the 10× images from Sections 1, 3, and 5 were observed simultaneously and the first 20 glomerular profiles present in all three slides were noted and numbered 1–20. The 100× lens was used to image the 20 glomerular profiles on each of the sections. For the WG method the 10× lens was used to imaged and identify the glomerular profiles present in Section 1 that were sequentially numbered 1–20. Using the labeled 10× image and 100× lens the 20 labeled profiles were imaged.

### 2.5 Measuring protocols

#### 2.5.1 Cavalieri method

The 100× images were viewed using Photoshop with window magnification of 67%. A grid of points 100 mm apart was superimposed over each profile image (Figure S2) from a glomerulus. The number of grid points falling on each profile from a glomerulus was counted. The volume of an individual glomerulus was calculated with the equation:

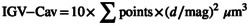

where 10 is the distance between sections in microns, ∑points is the sum of grid points falling on all the profiles from a glomerulus, *d* is the distance between grid points in microns, and mag is the magnification of the images. The magnification of the images was validated using a stage micrometer. The MGV‐Cav for an animal was calculated as the average of the 20 individual volumes.

#### 2.5.2 2‐Profile method

The volume of a sphere can be determined using two arbitrary parallel profiles from the sphere that are a known distance apart (Najafian et al., 2002). For this method glomeruli must be assumed to be spheres and thus the two glomerular profiles are assumed to be circles. The 100× images were viewed with Photoshop. The same grid of points used for the Cav method was superimposed over each image. The number of points falling on each profile was counted and recorded. The distance between grid points was divided by the image magnification, then squared, resulting in the area represented by one grid point. The area of each glomerular profile was calculated by multiplying the number of grid points falling on a profile by the area in microns represented by one grid point. Assuming the profiles were circles, the radii of the two profiles were calculated. The individual GV (IGV‐2P) were then calculated using the equation:

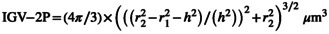

where *r*_1_ is the radius of the smaller glomerular profile, *r*_2_ is the radius of the larger glomerular profile, and *h* is the distance in microns between sections. MGV‐2P for an animal was determined by calculating the average of the 20 individual volumes.

#### 2.5.3 3‐Profile method

To test whether using three profiles increases the accuracy and/or precision of the individual GV we introduce a novel 3P method here. First, the glomerular volume was calculated by the 2P method using images from Sections 1 and 3 for each of the 20 glomeruli, then a second volume was calculated using images from the same 20 glomeruli present in Sections 3 and 5. Finally, the individual glomerular volume (IGV‐3P) was determined by calculating the average of the two MGV‐2P volumes for each of the 20 glomeruli. The MGV‐3P for an animal was then determined by calculating the average of the 20 individual volumes.

#### 2.5.4 Weibel–Gomez method

The WG method also assumes the glomeruli are spheres (Weibel, 1979). This method cannot measure IGV; it can only find the MGV of the measured glomeruli. A random number generator was used to determine which profile from each glomerular image stack was used (Figure S2). The number of points counted for that profile from the Cav stack was used to calculate the mean glomerular volume (MGV‐WG):

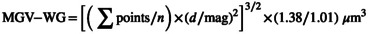

where ∑points is the sum of grid points falling on all the glomerular profiles, *n* is the number of profiles measured, *d* is the distance between grid points in microns, mag is the magnification of the images, 1.38 is the shape factor assuming the glomeruli are spheres, and 1.01 is correction factor assuming a CV of 0.10 for the glomeruli within the kidney.

### 2.6 Time intensiveness

The time needed to complete each step of the protocols per kidney, for embedment, sectioning, imaging, and measuring was noted for each of the four measuring methods (Table S1).

### 2.7 Magnification of images

Images obtained using the 100× lens are considered the gold standard, but it takes less time to locate and focus the glomeruli than when using lower magnification lenses. To test if a lower resolution lens would be more efficient, glomeruli embedded in plastic and previously measured using the 100× images were reimaged with the 40× lens and measured with the different methods. The spacing of grid points was set at 40 mm since the areas of the profiles were 40% less than when using the 100× lens. Thus, approximately the same number of points fall on the same profile whether using the 100× or 40× lens resulting in similar precision due to point counting.

### 2.8 Statistical analyses

#### 2.8.1 Tests of accuracy

We assessed the concordance between gold standard Cav glomerular volume measurements and alternative measurements via Lin's concordance correlation coefficient (Lin, 1989). This metric combines both precision and accuracy and increases (to a maximum of 1) as the line of best fit between two sets of measurements approaches the line of unity as the observed data skew more tightly to that line. We used linear regression to determine the equation of a line that would best relate measurements from alternative assessments to the gold standard. We compared MGV obtained between techniques using the unpaired *t*‐test. Two‐tailed *p*‐value of 0.05 was considered significant.

#### 2.8.2 Tests of precision

While all morphometric data are limited by the biological variability inherent in each sample (FSGS> controls as reported) (Banu et al., 2021), in our study it was restricted to 20 glomeruli in each sample. We used CV to compare precision within each sample but across the four measurement methods.

#### 2.8.3 Bias

We evaluated bias in MGV estimation and IGV values by each method using Bland–Altman plots.

#### 2.8.4 Number of glomeruli

We also empirically tested the proportion of times sampling 10 random glomeruli would result in a mean volume within 5%, 10%, or 25% of the model mean, that is, mean volume obtained by sampling 20 glomeruli. For this analysis, we randomly sampled 5, 10, or 15 glomeruli from each mouse, calculated mean glomerular volume of the sample, and determined whether the mean volume of these sampled glomeruli was within 5%, 10%, or 25% of the model mean. We then repeated this random sampling procedure for a total of 1000 iterations and reported the proportion of times the mean obtained was within 5%, 10%, or 25% of the model mean. We report logit‐transformed 95% confidence interval of this proportion by clustering at the level of the animal (Dean & Pagano, 2015). We present these data for all mice as well as based on whether the underlying diagnosis was FSGS or control. In this analysis we only included animals with 20 glomeruli sampled (*n* = 14). All analyses were conducted in Graphpad Prism.V9 and STATA (version 14.2).

## 3 RESULTS

### 3.1 Samples used for study

**FIGURE 1:**
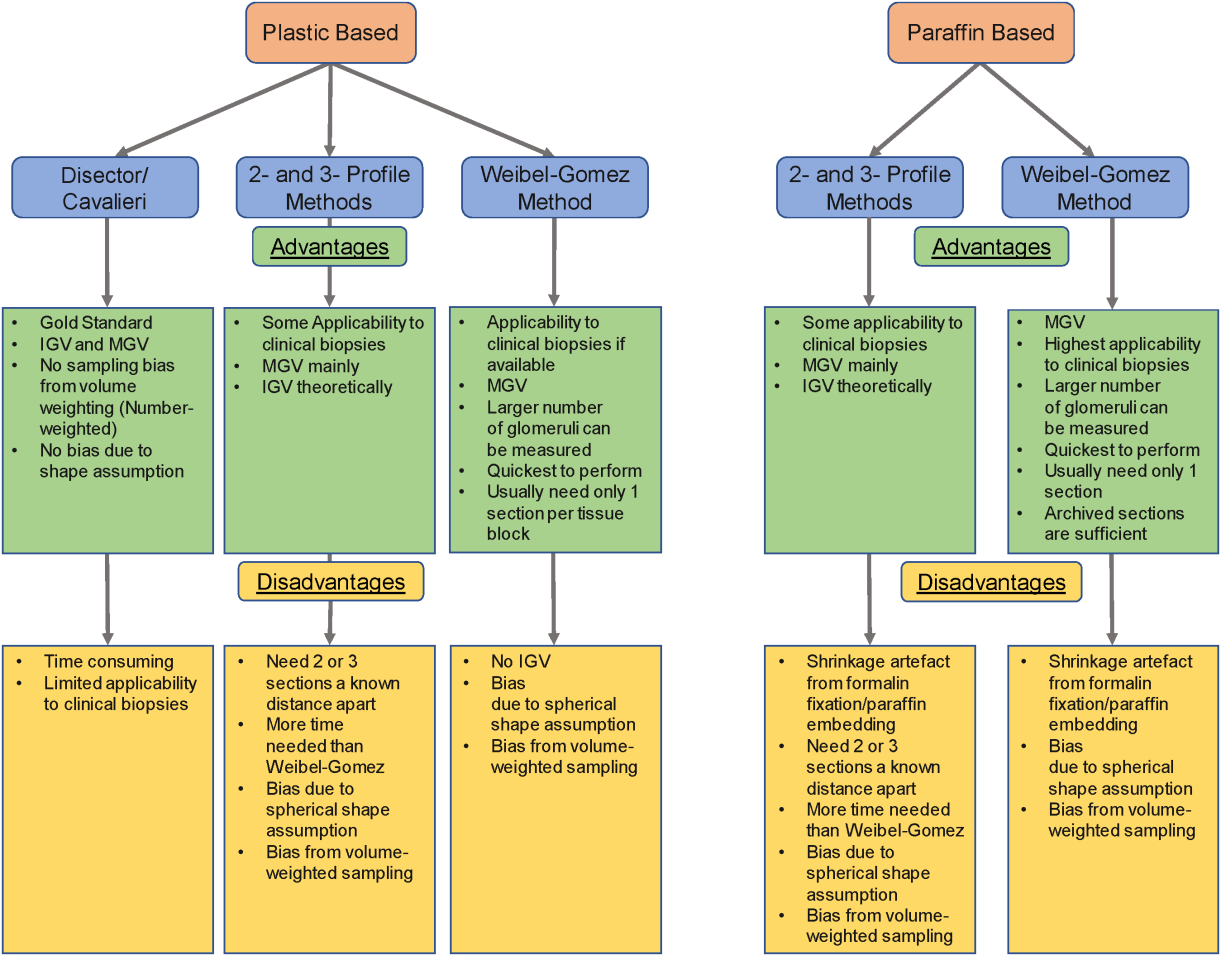
Flow diagram of advantages and disadvantages of the four morphometric techniques in plastic‐ and paraffin‐embedded tissues.

To evaluate GV during significant glomerular injury we used BALBc/J (*n* = 10) mice from two FSGS models of hypertrophic injury and a previously published aging model (Banu et al., 2021; Wei et al., 2018). The mice in these FSGS models had confirmed podocyte loss, glomerulosclerosis, proteinuria, and azotemia. In each animal, we evaluated three morphometric methods against the gold standard Cav method in both plastic‐ and paraffin‐embedded tissues using the 100× images. We evaluated sample size using up to 20 individual glomeruli per animal, to generate GV‐Cav. We modified the previously described 2P method (Najafian et al., 2002), and evaluated the inclusion of a third glomerular profile using an additional sequential tissue section (i.e., 3P method). Figure 1 illustrates the advantages and disadvantages of the four methods in plastic‐ and paraffin‐embedded tissues. For instance, like the Cav method, the 2P or 3P methods provided IGV and MGV, while the WG method can only provide MGV. The latter three methods assume spherical shape of glomeruli (incorporating a bias), while Cav method is shape independent. The average time‐intensiveness of each method is tabulated in Table S1.

### 3.2 Empiric validation of calculating mean volume from 5‐, 10‐, and 15‐ glomeruli samples in controls versus FSGS

Using MGV‐Cav on either plastic or paraffin tissue, prior work reported lack of significant additional benefit in MGV estimation above 10 glomeruli sampled per specimen (Najafian et al., 2002; Puelles et al., 2012). Here, we empirically tested the hypothesis that the MGV obtained by sampling 5, 10, and 15 glomeruli closely matches the mean obtained by sampling 20 glomeruli. By GV‐Cav, sampling greater than 20 glomeruli per sample is highly time intensive (Table S1), and unusual in most experimental data. We used IGV and calculated MGV from 1000 iterations of random sampling of 5, 10, or 15 glomeruli among 20 glomeruli. In modeling 5‐glomerular samples, among FSGS mice we noted that sample mean was within 5% of model mean only 32% of the time, versus 41% of the time in control animals (Table 1). When sampling 10 glomeruli, 84% (range 76–90) of the mean volumes obtained was within 10% of the model mean, 100% was within 25% of model mean, whereas only 53% (range 43–62) was within 5% of the model mean. Hence, we report the need to sample at least 10 glomeruli so that the observed mean would be within 10% of model mean in the majority (~80%) of cases. Sampling 15 glomeruli was needed to achieve the observed mean within 5% of model mean in 80% of cases.

**TABLE 1:** Proportion of times mean volume obtained by sampling 5 or 10 glomeruli was within given range of that obtained by sampling 20 glomeruli.

Magnification	Within given % of model mean)	Glomeruli sampled	Overall (*n* = 20)	FSGS (*n* = 10)	Controls (*n* = 10)
40×	5	5	36 (29, 44)%	32 (26, 39)%	40 (26, 55)%
40×	5	10	56 (47, 65)%	51 (42, 61)%	61 (43, 77)%
40×	5	15	80 (72, 86)%	76 (67, 84)%	83 (67, 92)%
40×	10	5	63 (53, 71)%	59 (49, 68)%	66 (47, 82)%
40×	10	10	84 (77, 89)%	83 (74, 89)%	85 (70, 93)%
40×	10	15	98 (96, 99)%	97 (94, 99)%	98 (96, 99)%
40×	25	5	95 (93, 97)%	95 (90, 97)%	96 (91, 99)%
40×	25	10	100 (100, 100)%	100 (100, 100)%	100 (100, 100)%
40×	25	15	100%	100%	100%
100×	5	5	37 (29, 46)%	32 (25, 40)%	42 (27, 58)%
100×	5	10	59 (50, 68)%	54 (44, 63)%	64 (46, 79)%
100×	5	15	80 (73, 86)%	78 (69, 85)%	82 (66, 92)%
100×	10	5	64 (54, 73)%	60 (50, 70)%	67 (47, 83)%
100×	10	10	85 (78, 90)%	84 (76, 90)%	85 (70, 93)%
100×	10	15	98 (97, 99)%	98 (96, 99)%	98 (96, 99)%
100×	25	5	96 (93, 97)%	95 (91, 97)%	96 (91, 98)%
100×	25	10	100 (100, 100)%	100 (100, 100)%	100 (100, 100)%
100×	25	15	100%	100%	100%

*Note*: 1000 iterations of random sampling of 5 or 10 glomeruli. Only includes those with 20 glomeruli. Model mean = mean glomerular volume obtained by sampling 20 glomeruli.

**FIGURE 2:**
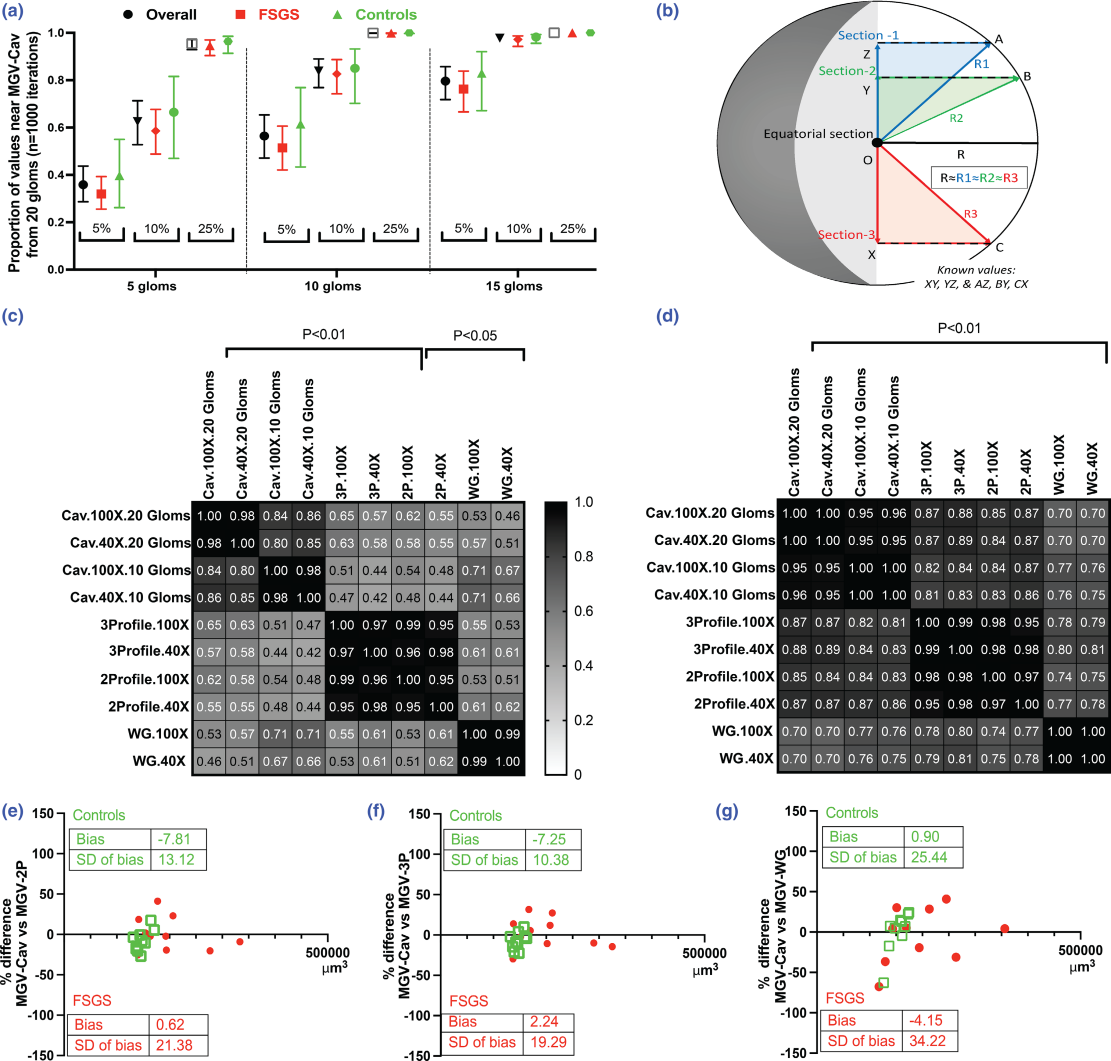
Evaluation of methods to estimate mean glomerular volume. (a) Plot shows mean ± 95% CI limits of estimates of accuracy of 5‐, 10‐, or 15‐glomerular samples versus 20‐glomeruli samples in control, FSGS, and overall samples using MGV‐Cav. (b) Schematic illustrates the 3‐profile method. R1, R2, and R3 represent three radii obtained from sections through the potential glomerular sphere. (c, d) Heat maps show correlation matrices of Spearman and Pearson correlation coefficients, respectively, of plastic‐based MGV by different methods used here versus MGV‐Cav (20 glomeruli; 100×). Square brackets show *p*‐values of correlation in each. (e–g) Bland–Altman plots show the bias observed in MGV in each animal sample when comparing MGV‐Cav versus (e) 2‐profile (f) 3‐profile (g) Weibel–Gomez methods, respectively. Inlay shows mean and SD of bias in each comparison for controls and FSGS mice.

In general, we noted that controls tended to have observed mean within a given percentage of model mean in a greater number of iterations than those with FSGS; however, no significant differences in these proportions were obtained when evaluating our FSGS or control animals separately (Figure 2a). Indeed, in both control and FSGS animals, 15‐glomerular samples were highly reflective of 20‐glomerular model MGV. Hence using our mouse models, these data provide a quantitative estimate of precision for MGV obtained from 10 IGVs when using GV‐Cav. We noted that we needed to sample at least 10 glomeruli so that the observed mean would be within 10% of model mean in 80% of cases. On the other hand, we would need to sample 15 glomeruli to achieve observed mean within 5% of model mean in 80% of cases.

### 3.3 Comparison of mean glomerular volume measurements in plastic‐embedded tissue in control and FSGS mice

Our IGV data suggested that in the GV‐Cav method, 10 glomeruli provided a reasonable estimate of MGV as obtained from 20 glomeruli (Figure 2a). Here we evaluated the relationships of MGV obtained by each plastic‐based method with MGV‐Cav (20 glomeruli 100× images; sampled glomeruli per animal of 3P, 2P, WG = 10.8 ± 2.6; median = 10). Figure 2b shows a schematic of the 3P method introduced here, which builds upon the published 2P method and uses an additional intersecting section (Section 3) at known distance from Sections 1 and 2 to calculate the radius of a given glomerulus. We first compared correlations between MGV‐Cav and MGV by the other methods and simultaneously evaluated each when section images were captured using two magnifications (40× vs. 100×). As shown in heatmaps in Figure 2c,d (Spearman‐ and Pearson‐ correlation matrices, respectively), each MGV measurement correlated significantly with MGV‐Cav. MGV‐3P and MGV‐2P (40× or 100×) were highly correlated with each other, and both significantly correlated with MGV‐Cav with higher correlation coefficients versus MGV‐WG. MGV‐3P showed inconsistent benefit in correlations compared to MGV‐2P at either magnification. On average, MGV from images obtained by 100× showed higher correlations over 40× images; however, within the Cav method 40× and 100× image‐based MGV showed similar correlations with the gold standard Cav method. While correlations can reveal relationships between data points, we next evaluated accuracy using Lin's concordance coefficient. Here we studied control and FSGS animals separately. Table 2a shows Lin's concordance values between MGVs obtained by each method compared to GV‐Cav in control animals. Concordance between MGV‐WG was markedly lower than obtained by all other plastic based techniques. By each method evaluated, differences in image magnifications (40× vs. 100×) did not consistently or beneficially alter obtained concordance values (data not shown). Furthermore, applying our novel 3P method showed an incremental but small improvement in Lin's concordance versus the 2P method in control animals.

**TABLE 2a:** Lin's concordance correlation coefficient (SE) compared to gold standard mean volume in all 20 mice. Methods reflect the mean of 20 glomeruli in plastic except Weibel‐Gomez method is in paraffin.

Method	Concordance correlation
Cavalieri 100×	0.935 (0.026)
Cavalieri 40×	0.934 (0.025)
2‐profile 100×	0.818 (0.076)
2‐profile 40×	0.836 (0.070)
3‐profile 100×	0.860 (0.061)
3‐profile 40×	0.853 (0.061)
Weibel‐Gomez method 100×	0.692 (0.119)
Weibel‐Gomez method 40×	0.689 (0.119)

We then similarly evaluated concordance between MGVs obtained by other plastic‐based methods in FSGS animals versus MGV‐Cav shown in Table 2b. Except in the WG method, a high concordance was observed in MGVs in Cav, 2P, or 3P method when compared to the gold standard Cav method. Similar to observations in control animals, increased image magnification for measurements had inconsistent benefit to MGV by all techniques. On the other hand, the 3P method again showed an incremental benefit in concordance values over the 2P method. Table 2c displays regression equations that relate MGVs obtained using 2P or 3P or WG methods (40× or 100× images) to the MGV‐Cav.

**TABLE 2b:** Lin's concordance correlation coefficient (SE) compared to gold standard mean volume in 10 FSGS mice. Methods reflect the mean of 20 glomeruli in plastic except Weibel‐Gomez method is in paraffin.

Method	Concordance correlation
Cavalieri 100×	0.947 (0.027)
Cavalieri 40×	0.944 (0.026)
2‐profile 100×	0.804 (0.115)
2‐profile 40×	0.830 (0.104)
3‐profile 100×	0.843 (0.094)
3‐profile 40×	0.840 (0.091)
Weibel‐Gomez method 100×	0.712 (0.169)
Weibel‐Gomez method 40×	0.693 (0.177)

**TABLE 2c:** Linear regression variable relating various methods (10.76 ± 2.56 glomeruli/animal; median = 10 using plastic methods) to the gold standard (10 glomeruli/animal MGV‐Cav). Includes FSGS and Control Mice (*N* = 10 each).

Method	Slope	Intercept
Cavalieri 100×	0.796	31,400
Cavalieri 40×	0.778	36,100
2‐profile 100×	0.912	21,000
2‐profile 40×	0.903	22,700
3‐profile 100×	0.937	14,900
3‐profile 40×	0.999	7500
Weibel‐Gomez method 100×	0.587	71,500
Weibel‐Gomez method 40×	0.575	74,700

In Bland–Altman plots to estimate bias in MGV data obtained from these comparisons (Figure 2e,f), we observed that MGV‐3P and MGV‐2P tended marginally to underestimate MGV‐Cav in control animals (~7%), with a lower SD of bias in MGV‐3P. WG showed larger spread in values and inconsistent bias (Figure 2g). Importantly, FSGS animals tended to have higher MGVs and showed greater spread of bias than controls by each method.

### 3.4 Comparison of individual glomerular volume measurements in plastic‐embedded tissue of control and FSGS mice

Next, we focused on IGV measurements comparing IGV‐Cav to IGV‐2P and IGV‐3P methods aiming to evaluate accuracy, precision, and bias. The GV‐WG method cannot report IGV and was not included in this analysis. Here, we evaluated IGV using all the glomeruli from each animal that overlapped among these three methods for IGV assessment from control (6 animals, 57 glomeruli; 9.2 ± 2.2) and FSGS (10 animals, 91 glomeruli; 9.0 ± 1.9 glomeruli/animal). We also tested both 100× and 40× magnification images for these comparisons, where the former is typically used. The third section in IGV‐3P allowed for the calculation of an additional GV‐2P for each sampled glomerulus (Figure 2b).

**FIGURE 3:**
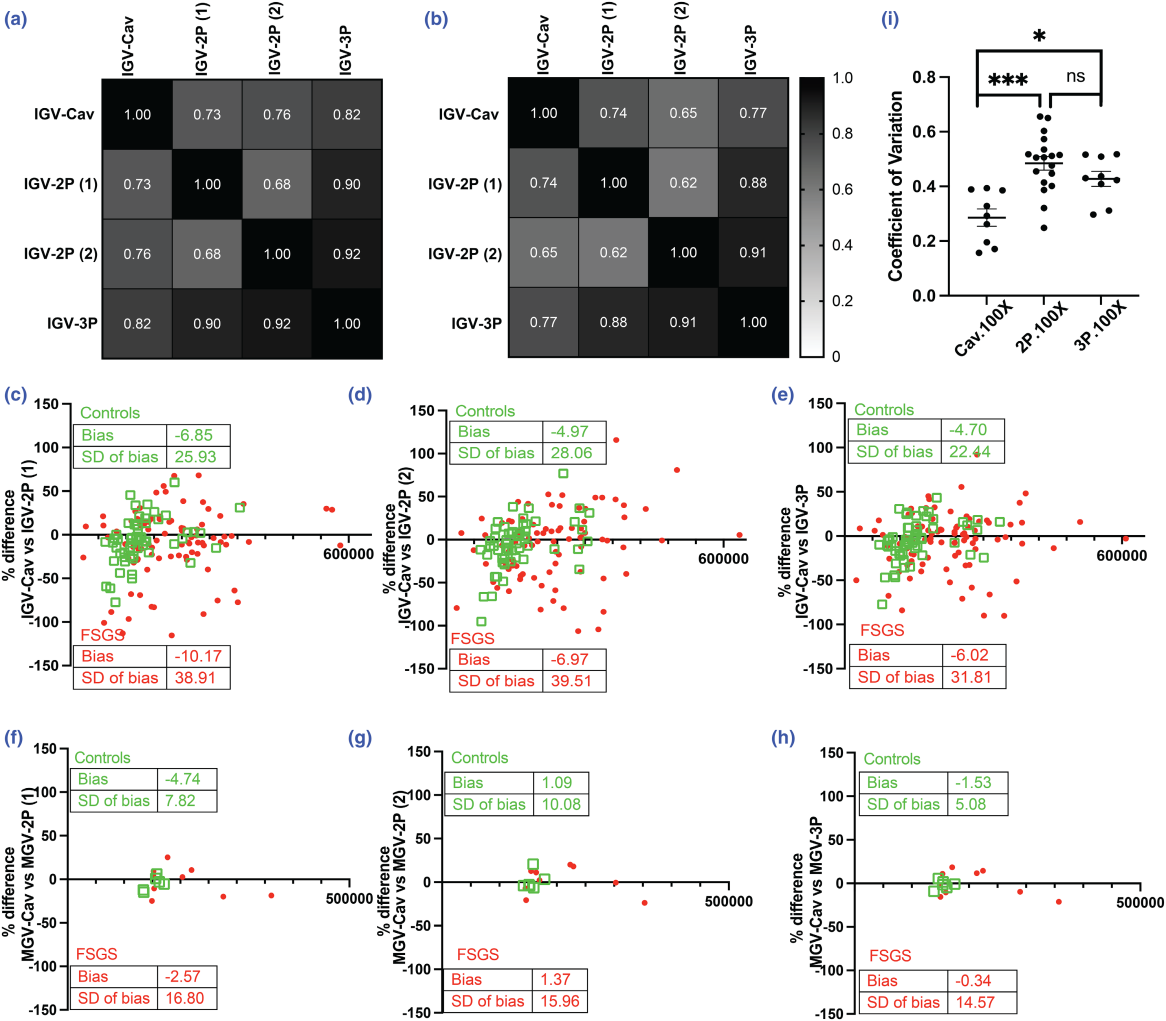
Evaluation of methods to estimate individual glomerular volume. (a, b) Heat maps show correlation matrices of Spearman and Pearson correlation coefficients, respectively, of plastic‐based IGV by different methods used here versus IGV‐Cav (20 glomeruli; 100×). All these coefficients had *p*‐values <0.05. (IGV‐2P (1) & (2) annotate two separate IGV‐2P measurements obtained from the three profiles that are available in each glomerulus when generating IGV‐3P). (c–e) Bland–Altman plots show the bias observed in IGV in each glomerulus from Control and FSGS samples when comparing IGV‐Cav versus (c) IGV‐2P (1) (d) IGV‐2P (2) (e) 3‐profile methods, respectively. (f–h) Bland–Altman plots show the bias observed in IGV in each glomerulus from control and FSGS samples when comparing IGV‐Cav versus (c) IGV‐2P (1) (d) IGV‐2P (2) (e) 3‐profile methods, respectively. Inlay tabulates mean and SD of bias in each comparison for controls and FSGS mice. (i) Dot‐plot and error bars (mean ± SEM) plot coefficient of variation obtained from IGV measurements (9.0 ± 1.9 glomeruli/animal) within each of the 10 FSGS animals. *Kruskal–Wallis with post‐test Dunn's test *p* < 0.05. ***Kruskal‐Wallis with post hoc Dunn's test *p* < 0.001.

Overall, IGV measurements by the 3P and 2P methods were significantly correlated with IGV‐Cav by Pearson *r* and Spearman rho (*p* < 0.0001), in control and FSGS mice (Figure 3a,b). IGV‐3P method showed marginally improved correlations in these data (Figure 3a,b). We then evaluated concordance between IGVs measured by the three methods, while allowing for clustering at the individual animal level. Here again, the 3P method provided marginal benefit over the 2P method overall and in FSGS animals (4%–7%, Table S2A). Hence, our new 3P method again provided marginally improved accuracy of estimation of IGV measured by the gold standard Cav method. In control mice, Bland–Altman plots of IGV estimation revealed an underestimation bias (~4%) when using the 2P or 3P technique (Figure 3c–e), versus IGV‐Cav. Among FSGS mice, Bland–Altman analyses showed a consistent underestimation by MGV‐3P and MGV‐2P versus GV‐Cav but with wider standard deviations of bias (Figure 3c–e). We then evaluated bias of MGV calculated for each animal when only overlapping IGVs were included. We hypothesized this would minimize bias induced by biological variability between IGV among different glomeruli that were sampled for each technique and allow estimation of bias related to technical differences. Here, when only glomeruli that overlapped across all techniques were included for MGV, the means and SDs of the bias in each comparison were reduced (i.e., vs. MGV calculated in Figure 2). The greatest reduction in bias was observed for the 3P method among control animals, although these were not identified as significantly different (i.e., Figure 3f–h vs. Figure 2e–g; unpaired *t*‐test *p* > 0.05 for each comparison). While the biases were not significantly different, the concordance coefficients of MGV calculated by overlapping IGVs were significantly improved in the 2P and 3P methods, suggesting improved accuracy by each method when biological GV variation was removed (Figure S3; Table S2B).

Finally, we evaluated the CV of each technique within an animal as a marker of precision when sampling the same glomerular IGVs. Whereas the CV was not different between the three methods in control mice (data not shown), in FSGS mice IGV‐Cav had significantly lower CV than both 2P and 3P methods (Figure 3i).

### 3.5 Relating MGV from paraffin‐embedment to plastic‐embedment in control and FSGS animals

**FIGURE 4:**
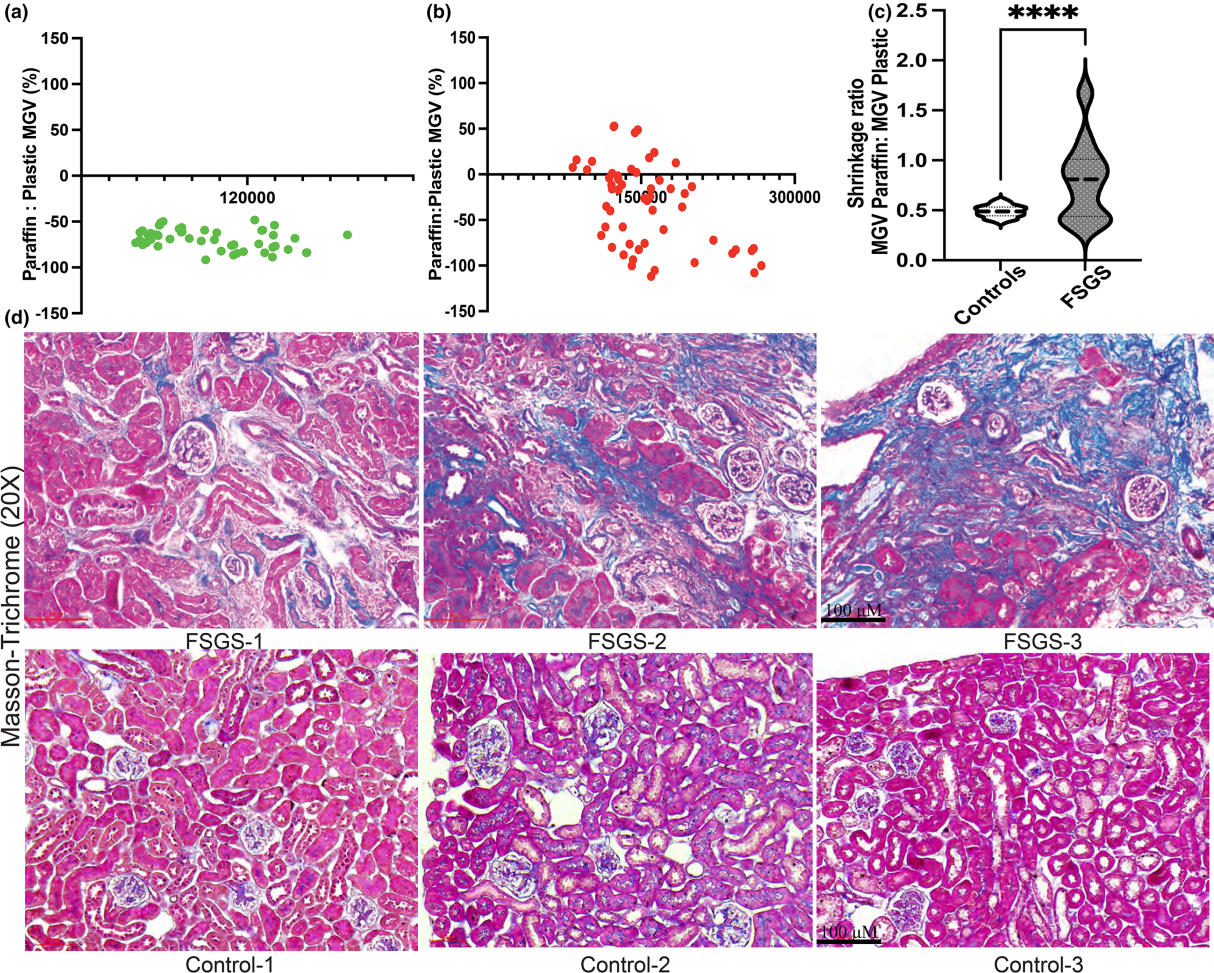
Differential shrinkage artifact and periglomerular/glomerular fibrosis in FSGS animals. (a) Bland–Altman plots show ratios between paraffin:plastic measurements of MGV obtained in each control animal from 10 or 20 glomeruli within respective techniques [11 comparisons from 6 control animals (green dots)]. (b) shows these same ratios of MGVs obtained from 10‐glomeruli in each FSGS animal [6 comparisons from 10 FSGS mice (red dots); X‐axis shows the corresponding MGVs]. (c) Violin plot (dashed line at median) summarizes the shrinkage artifact obtained from all measurements between FSGS and control animals [mean ± SEM; ****p < 0.0001]. (d) Photomicrographs (20×) from kidney sections of representative FSGS and control animals within our dataset showing variable glomerular fibrosis (area positive for Trichrome stain) in each.

While morphometry can be performed in plastic‐ or paraffin‐embedded tissue, formalin fixation and paraffin embedding are known to cause shrinkage with bias or distortion of morphometric measurements in other tissues (Ladekarl, 1994; Schneider & Ochs, 2014). Here we compared MGV measurements between 2P or 3P, and WG performed in plastic versus these same methods in paraffin tissue within each animal. In control animals, we identified a nearly uniform reduction in MGVs in paraffin‐embedded measurements versus corresponding plastic measurements (across all techniques mean ± SD ratios = 0.52 ± 0.06; mean of SDs across techniques is 0.11) This was reflected in the uniform bias identified in Bland–Altman plots of controls evaluating paraffin:plastic MGV ratio (Figure 4a). These findings are consistent with a shrinkage artifact with tissue processing for paraffin embedding. Contrastingly, FSGS animals showed lower mean reductions in MGV compared to corresponding plastic measurements via each technique (mean ± SD ratios = 0.78 ± 0.07; mean of SDs across techniques = 0.28). The greater variability in bias in FSGS animals is represented as a Bland–Altman plot (Figure 4b). Mean shrinkage was also significantly different between control and FSGS animals (Figure 4c). The variability of shrinkage within each FSGS animal when ratios were obtained from different techniques likely represented greater interglomerular IGV variation in kidneys with FSGS pathology. This was reflected in the CV in IGVs obtained in FSGS glomeruli as we previously reported (Banu et al., 2021). Moreover, there was marked variability in the paraffin to plastic MGV ratios between animals with FSGS. As expected, Masson's trichrome stain of FSGS animals showed glomerular fibrosis, albeit of varying extent (Figure 4d). These data suggest glomerular fibrosis which develops in FSGS glomeruli could restrict the shrinkage artifact in paraffin‐embedded MGV estimation.

We also recently reported increased MGV in mice models with FSGS compared to the respective controls in each model (Banu et al., 2021). Here we re‐evaluated the data given the contribution of reduced shrinkage artifact seen in FSGS glomeruli to the reported glomerulomegaly reported in FSGS. Table 3 compares MGVs in control and FSGS animals across plastic‐ and paraffin‐based techniques used here. As expected, significant glomerulomegaly in FSGS was clearly identifiable in paraffin‐based measurements where control glomeruli would be expected to have greater reduction in GV and MGV. In plastic‐based methods, MGV was increased in FSGS animals versus controls, albeit insignificantly in many comparisons. Hence glomerulomegaly reported in paraffin‐based morphometric studies of FSGS is likely a combination of true increases in GV, as well as a reflection of glomerular fibrosis and reduced shrinkage.

**TABLE 3:** Comparison of mean glomerular volumes of FSGS animals versus control animals using plastic‐based and paraffin‐based measurements.

Plastic		Control	FSGS	*t*‐test
		Mean ± SD	Mean ± SD	*p*‐value
20 Gloms	Cav‐100×	152,400 ± 10,070	198,900 ± 72,570	0.06
	Cav‐40×	150,900 ± 10,390	196,800 ± 71,950	0.06
10 Gloms	Cav‐100×	160,200 ± 17,880	202,100 ± 90,210	0.17
	Cav‐40×	159,000 ± 18,030	199,700 ± 93,140	0.19
	2‐profile‐100×	141,800 ± 20,380	197,200 ± 57,410	**0.01**
	2‐profile‐40×	142,100 ± 20,900	196,800 ± 60,350	**0.01**
	3‐profile‐100×	142,100 ± 14,930	201,000 ± 58,730	**0.007**
	3‐profile‐40×	141,300 ± 17,230	195,300 ± 55,370	**0.009**
	WG‐100×	156,800 ± 32,500	197,900 ± 86,250	0.18
	WG‐40×	152,500 ± 28,770	198,400 ± 88,450	0.14
**Paraffin**		**Control**	**FSGS**	***t*** **‐test**
		**Mean ± SD**	**Mean ± SD**	***p*** **‐value**
20 Gloms	2‐profile‐40×	70,100 ± 10,590	131,700 ± 30,660	**0.001**
	3‐profile‐40×	72,800 ± 11,680	133,400 ± 32,760	**0.002**
	WG‐40×	78,600 ± 14,770	115,500 ± 32,890	**0.04**
10 Gloms	2‐profile‐40×	69,500 ± 7060	120,300 ± 35,630	**0.009**
	3‐profile‐40×	71,600 ± 4910	123,500 ± 41,250	**0.02**
	WG‐40×	81,600 ± 20,410	123,900 ± 40,310	0.05

The bold values are the significant *p*‐values for the *t*‐tests.

Abbreviations: Cav, Cavalieri; FSGS, focal segmental glomerulosclerosis; WG, Weibel–Gomez method.

## 4 DISCUSSION

From experimental and clinical data, morphometric estimates of glomerular volume have diagnostic and prognostic implications, over and above information obtained from histology alone. The most accurate technique for GV estimation Cav/disector is time‐consuming to carry out, wastes tissue sections, and requires considerable expertise limiting its utility in retrospective clinical studies. Here we used tissue samples, both plastic and paraffin, from control and FSGS mice to understand relationships between GV obtained by Cav/disector technique and other reported techniques. In both paraffin and plastic, we also tested a novel 3P technique as a modification of the published 2P method, adding a third section to the 2P method with a goal to obtain a more precise estimation of the radius of the presumptive glomerular sphere. Within the gold standard GV‐Cav we also evaluated the precision of MGV estimation while using MGVs obtained from 10 IGV measurements versus 20 IGVs. In both FSGS and controls, we identified acceptable precision of 10‐glomerular MGV versus 20‐glomerular MGV within GV‐Cav. However, in 5‐glomerular sampling, ~40% (in FSGS) and ~30% (in controls) MGV measurements fell outside of the ±10% range of the model mean. In addition, while 5‐ or 10‐glomerular samples gave inferior estimates of 20‐glom MGV in FSGS than in controls, this disparity reduced in the 15 glomerular sampling data. In plastic‐based techniques, MGV‐2P or MGV‐3P measurements showed greater concordance with GV‐Cav than the GV‐WG method. MGV‐3P offered limited incremental benefit in correlation, concordance, and bias to the existing 2P method for IGV estimates but limited detectable benefit in MGV. Changing image magnification (100× vs. 40×) during points counting had minimum effect on MGV within the gold standard, GV‐Cav but had inconsistent effect when using other methods suggesting 100× should possibly be preferred in 2P, 3P, or WG. Finally, we identified a consistent reduction of GV values within control animals (~50%) when using paraffin‐based methods (vs. corresponding methods in plastic‐embedded tissue) demonstrating a clear shrinkage artifact due to tissue processing. Most interestingly, FSGS glomeruli showed significantly less but more variable shrinkage artifact likely due to glomerular fibrosis.

Our findings have implications for experimental and clinical morphometric studies. Our data provide a quantitative estimate of accuracy when 10 glomeruli are randomly sampled and MGV is obtained using GV‐Cav. This finding is consistent with prior reports (Najafian et al., 2002; Puelles et al., 2019). Here, other plastic methods, including the GV‐WG using 10‐glomerular samples, also provided acceptable accuracy in our analyses. This number of glomerular profiles was present in the majority of clinical biopsies from a recent dataset of human nephrotic syndromes from the NEPTUNE consortium (Puelles et al., 2019; Zhang et al., 2021). Overall, our data are reassuring regarding the acceptable and minimal observed technical variability in MGV both from overlapping and nonoverlapping IGV measurements by 2P or 3P techniques when performed by an expert. On the other hand, we identify that even within controls, biological variability between glomeruli predominates (Figure S3). Increased interglomerular GV variability has been reported in FSGS, and IGV‐Cav here showed a clear advantage in precision (significantly reduced CV) over 2P and 3P methods in FSGS. However, 10‐glomerular MGV showed similar concordance to 20‐glomerular MGV in our samples of both FSGS and control animals despite limitations (Figure 1).

Next, the spherical shape assumption in the 2P and 3P methods led to an underestimation bias in IGV and MGV, consistently detectable in control animals. The regression equations developed here could be applied to increase the accuracy and correct bias of IGV and MGV by 2P or 3P methods. The 2P or 3P methods when performed in plastic also showed high concordance with GV‐Cav in FSGS and controls, suggesting that these methods provided reasonable estimates of MGV when compared with GV‐Cav, and could be used for small animal studies. On the other hand, the increased precision of GV‐Cav occurs at the cost of the time‐intensiveness, that is twofold more intensive versus 3P, and threefold versus 2P in our analyses. Finally, while GV‐WG was inferior to both 2P and 3P methods, it is the least time‐intensive. Additionally, the GV‐WG method could likely remain useful in the scenario of multicenter clinical studies when there is limited accessibility to plastic‐ or paraffin‐embedded samples between the study centers, but relatively easy access to scanned whole‐slide images of biopsies. Along these lines, the explosion of multicenter clinical studies using automated digital morphometry have been overlaid on the WG equation (Barisoni et al., 2013; Nast et al., 2015; Zee et al., 2022).

Our data also provide an insight into the relationship between embedment and GV. While plastic‐embedment methods provided acceptable accuracy in control and FSGS animals, we identify clear differences between controls and FSGS pathology in paraffin‐embedment. To our knowledge, our findings also quantify for the first time the shrinkage artifact in GV estimation when using paraffin‐based methods. Hence studies estimating GV from paraffin need to account for shrinkage before reporting GVs in comparative animal data evaluating glomerular morphometry. This shrinkage (or lack thereof) would influence GV assessment, especially when significant and/or variable glomerular fibrosis is identified or expected. In these circumstances, comparative morphometric studies should aim to collect plastic‐embedded tissue. On the other hand, if both plastic‐ and paraffin‐embedded GV are available, the presence of less‐than‐expected shrinkage in paraffin could signify the presence of glomerular fibrosis in animal models. In humans, a greater amount background glomerular fibrosis may be expected with aging (Denic et al., 2019; Yang & Fogo, 2014), and the relevance of our findings will need to be specifically tested in this context.

We limited our analysis to BALBc/J mice versus other backgrounds, which could be considered a limitation. Larger glomeruli close to the medulla or adjacent to large blood vessels were excluded where possible in all techniques although not universally. Our goal was to preferentially sample cortical glomeruli and avoid sampling larger juxtaglomerular glomeruli. On the other hand, since only glomeruli that intersected the first section were used, there may have existed a bias toward larger glomeruli within cortical glomeruli. We also acknowledge that the estimate of the shrinkage artifact we report here could be influenced by biological and technical variations introduced by tissue processing.

In summary, we compare commonly applied glomerular morphometric techniques using both control and FSGS animals with plastic and paraffin tissue data. We quantify the accuracy of 5‐, 10‐, 15‐glomerular MGV in controls or FSGS, and develop relating equations between less‐intensive plastic‐based measurements to the gold standard Cav method. Our analyses reiterate the increased precision of the more intensive GV‐Cav in animal data but show acceptable accuracy and bias with the 2P and 3P methods. We demonstrate clearly and quantify the shrinkage bias during tissue processing for paraffin‐embedding and report the role of FSGS with resultant glomerular/periglomerular fibrosis in altering the shrinkage artifact, a phenomenon that needs to be considered in morphometric studies. Our findings have application to clinical and experimental studies using glomerular morphometry.

## AUTHOR CONTRIBUTIONS

FPW and MCM conceived and oversaw the overall study. ACR, QL, JMB, and MCM wrote the manuscript. JMB, ACR, QL, KB, and HS performed the experiments. JMB performed all the morphometry measurements. MCM and JCH conceptualized the workflow. ACR, QL, JMB, HS, AV, and JP contributed to the data generation, compiling, and illustration. DM and FPW performed statistical data analysis and interpretation. SP assisted in interpreting pathology. All authors contributed to the editing of the manuscript.

## FUNDING INFORMATION

MCM received research support from NIH R01DK122164 and R01DK132274. FPW received research support from NIH R01DK11319 and R01HS027626. This research was further supported by P30DK079310.

## CONFLICT OF INTEREST STATEMENT

No conflicts of interest, financial or otherwise, are declared by the authors.

## ETHICS STATEMENT

All animal procedures were conducted according to the IACUC‐approved protocol (# 2021‐20,363) at Yale University school of Medicine.

## Supporting information


Figure S1:

Figure S2:

Figure S3:

Table S1:
