# Supplementary figures and images for: Comparative evaluation of glomerular morphometric techniques reveals differential technical artifacts between focal segmental glomerulosclerosis and normal glomeruli
Source: Physiol Rep. 2023 Jul 9;11(13):e15688. doi: 10.14814/phy2.15688 (PMC10329935; doi:10.14814/phy2.15688)

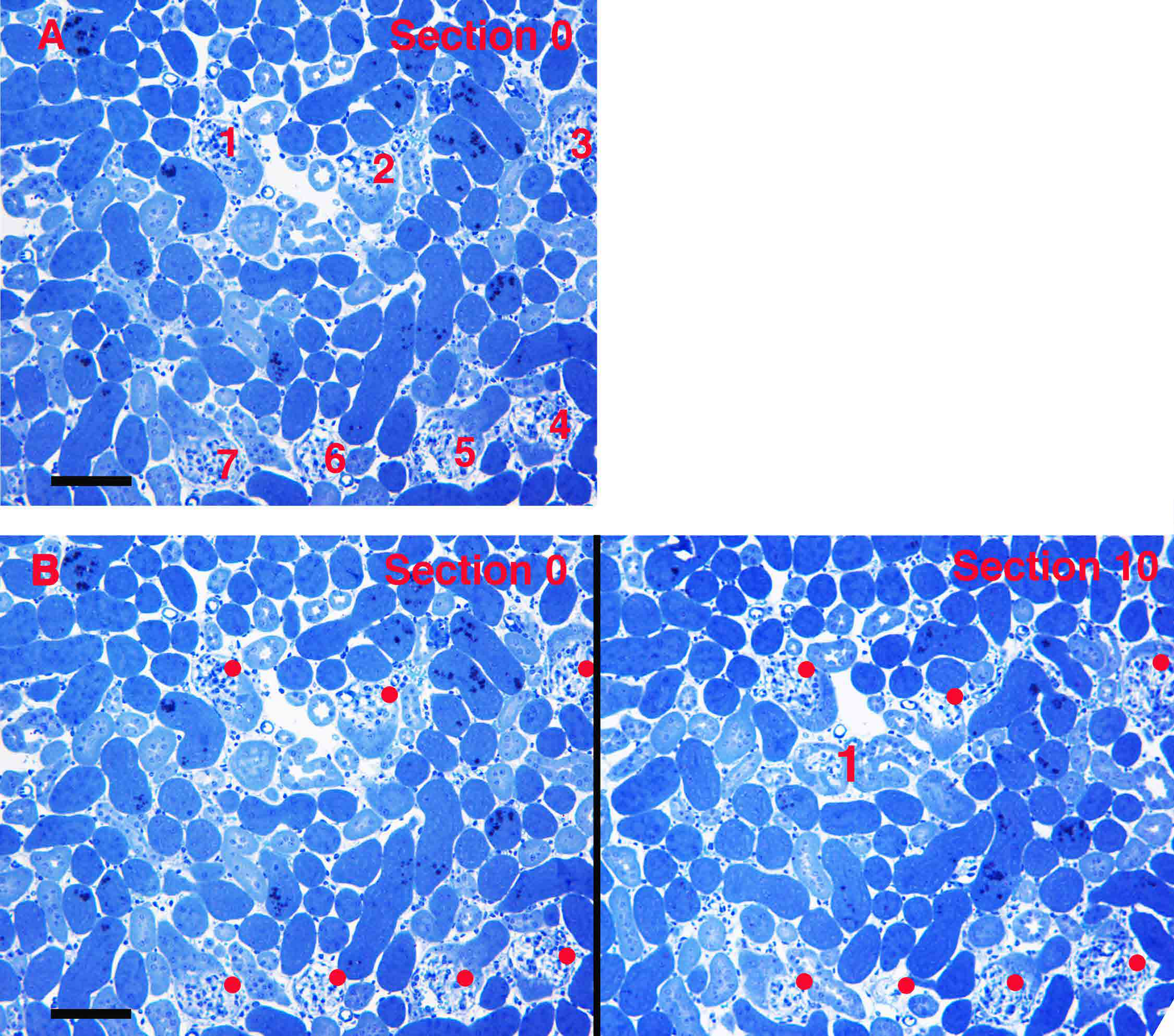

Supplement: Supplementary file 1 — Figure S1: [file PHY2-11-e15688-s002.tif]

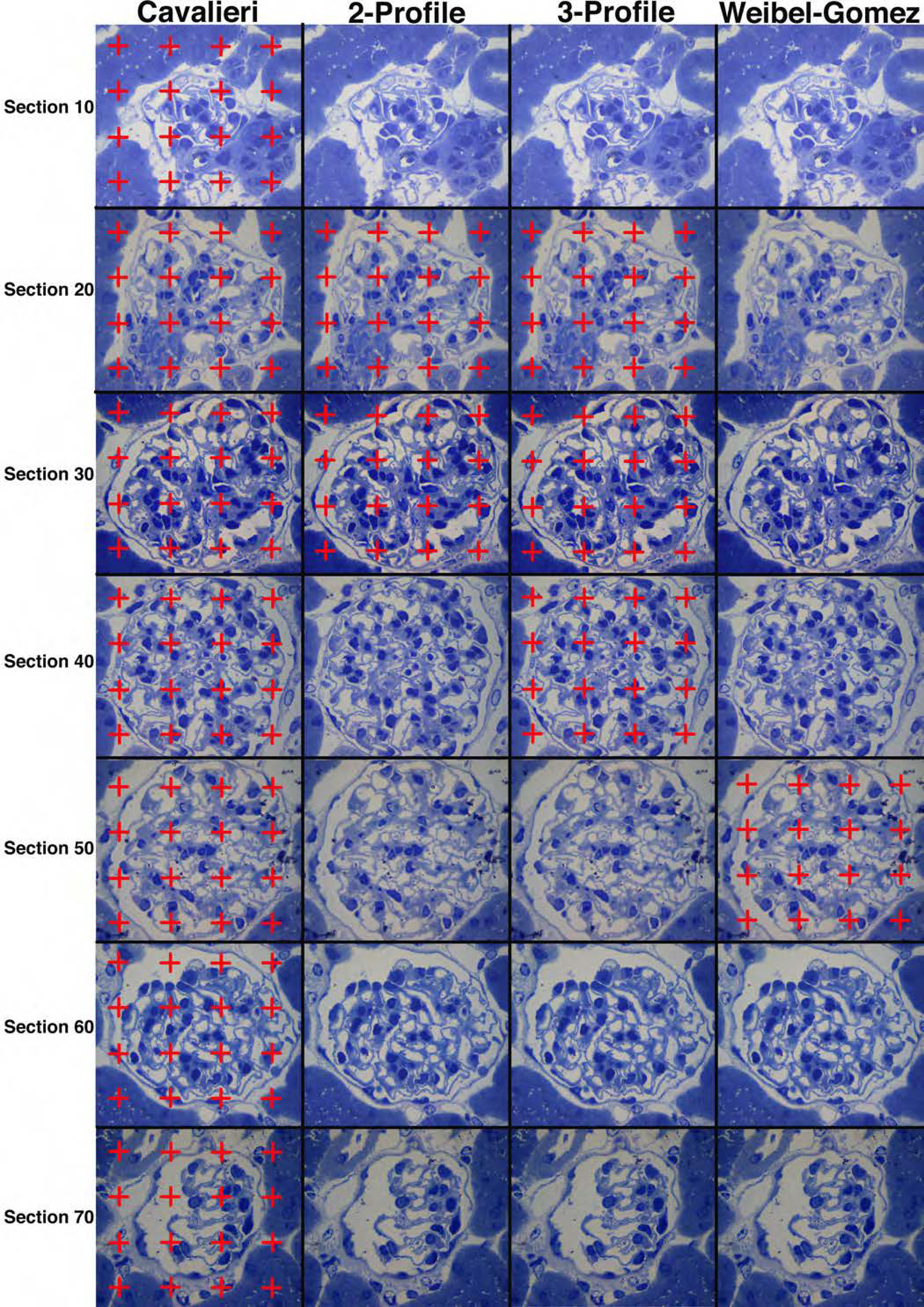

Supplement: Supplementary file 2 — Figure S2: [file PHY2-11-e15688-s001.tif]

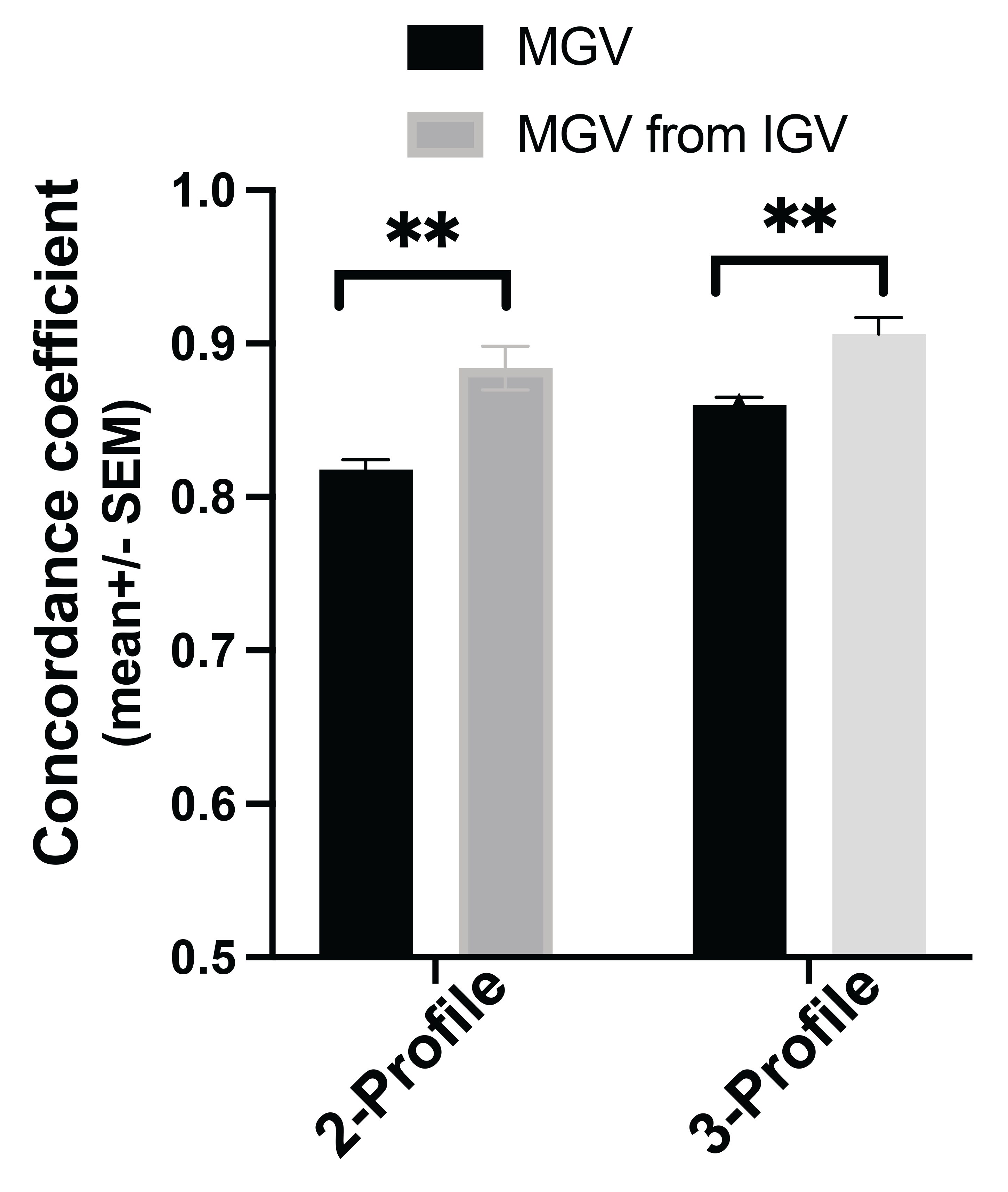

Supplement: Supplementary file 3 — Figure S3: [file PHY2-11-e15688-s003.tif]
