# Supplementary material for: Comparative evaluation of glomerular morphometric techniques reveals differential technical artifacts between focal segmental glomerulosclerosis and normal glomeruli
Source: Physiol Rep. 2023 Jul 9;11(13):e15688. doi: 10.14814/phy2.15688 (PMC10329935; doi:10.14814/phy2.15688)
Supplement: Supplementary file 4 — Table S1: [file PHY2-11-e15688-s004.docx]

**Table S1 Time intensiveness of plastic and paraffin-based techniques used for GV.**

|  | Amount of time per animal (in minutes for 10 gloms/animal) to process experimental steps | | | | | | | | |
| --- | --- | --- | --- | --- | --- | --- | --- | --- | --- |
|  | Plastic Embedment* | | | |  | Paraffin Embedment* | | |  |
| Procedure | Cavalieri | 2P | 3P | W-G |  | 2P | 3P | W-G |  |
| Section + Stain | 55 | 12 | 16 | 10 |  | 55 | 55 | 55 |  |
| Image | 67 | 24 | 37 | 13 |  | 28 | 38 | 21 |  |
| Measure | 32 | 11 | 16 | 6 |  | 11 | 15 | 6 |  |
| Total | 154 | 47 | 69 | 29 |  | 94 | 108 | 82 |  |

**Times for dehydration, and plastic or paraffin embedding are not included and are usually done by automated procedures overnight.*

**Table S2A Lin’s concordance correlation coefficient (SE) vs gold**

**standard for individual glomerular volume measurements (N=148**

**glomeruli, 15 mice). SE accounts for clustering at level of the mouse.**

|  | Method | Concordance Correlation (SE) |
| --- | --- | --- |
| Overall | 2-Profile-1 | 0.38 (0.09) |
|  | 2-Profile-2 | 0.34 (0.09) |
|  | 3-Profile | 0.41 (0.10) |
|  | | |
| FSGS | 2-Profile-1 | 0.41 (0.13) |
|  | 2-Profile-2 | 0.37 (0.12) |
|  | 3-Profile | 0.45 (0.13) |

**Table S2B Lin’s concordance correlation coefficient (SE) vs gold standard for MGVs obtained from individual glomerular volume measurements of overlapping glomeruli (N=15 mice). SE accounts for clustering at the level of the mouse.**

|  | 2-Profile-1 | 2-Profile-2 | 3-Profile |
| --- | --- | --- | --- |
| Overall (N=16) | 0.886 (0.05) | 0.883 (0.06) | 0.906 (0.042) |
| FSGS (N=10) | 0.867 (0.074) | 0.869 (0.083) | 0.887 (0.064) |
